# Alternative Allied Health Care Providers: Athletic Trainers Educating Stakeholders to Improve HPV-Related Infections

**DOI:** 10.1177/21501319251351080

**Published:** 2025-06-29

**Authors:** Justin Ceasar, Eva M. Frank, Rebecca Hall, Cole Hartert, Cayce Onks

**Affiliations:** 1 Allegheny Health Network, Pittsburgh, PA, USA; 2 Lebanon Valley College Athletic Training Department, Annville, PA, USA; 3 Columbia Borough School Department Athletic Training, PA, USA; 4 Oxford Area School District Athletic Training, PA, USA; 5 Penn State Health, Hershey, PA, USA

**Keywords:** athletic training education, human papillomavirus, vaccination rates, sports medicine, athletic trainers

## Abstract

**Context:**

Human Papillomavirus (HPV) has one of the lowest vaccination rates. Sports medicine providers are well suited to educate stakeholders about HPV-related infections, but athletic trainers (ATs) historically haven’t been actively involved in the prevention of this disease. This study aimed to examine ATs’ knowledge of HPV and their perceived role and responsibility in educating stakeholders about HPV.

**Methods:**

A virtual cross-sectional survey was sent to participants through the National Athletic Trainers’ Association data collection service. The survey consisted of a consent form, demographics, knowledge, and perception-based questions. An optional open-ended section was included.

**Results:**

Despite a lower response rate, 460 ATs participated in the study. Only 46% of all ATs scored above 70% on the knowledge-based questions. Those with previous HPV-related training displayed significantly more knowledge (average score of 77.5%) versus those without (64%). Those with training also felt that ATs are responsible for assisting with athletes’ general medical conditions (*P* = .007). ATs previously offered (*P* = .0001) or having received the HPV vaccine (*P* < .0001) had significantly more knowledge.

**Conclusions:**

ATs are well-positioned to educate stakeholders about HPV. Although knowledge levels are low, ATs perceived a responsibility to educate stakeholders directly contributing to preventing HPV from spreading.

The Human Papillomavirus (HPV) is a common sexually transmitted infection (STI) that can lead to cancer.^1^ In the United States (US), 1 in 4 people are currently infected with HPV.^1^ In 2018, 43 million HPV infections affected people in their late teens and early 20s.^2^ HPV spreads through sexual contact (vaginal, anal, or oral) with someone who is infected. Carriers of HPV may not always have signs or symptoms; therefore, any sexually active individual is at risk of contracting HPV.^2^ Subsequent health problems may not manifest until years after contracting the virus.^2^

In the US, over 30 000 people develop cancer annually as a result of an HPV infection.^1^ In women, the most common cancer to develop from HPV is cervical cancer.^3^ About 12 000 women annually are diagnosed with cervical cancer and about 4000 women die from complications related to the disease.^4^ HPV-related cancers are not as common in men but may develop as oropharyngeal, penile, and anal cancer.^2^ Fortunately, vaccines have been developed that are effective in the prevention of HPV-related infections and decreasing cancer incidence.^5^

HPV vaccines are among the most effective prophylactic vaccines available and represent the first vaccination to prevent infection by a mucosotropic sexually transmitted infectious agent.^6^ The vaccine is recommended to individuals up to age 26 years with consideration to age 45 years if benefits outweigh risks.^7^ It is important to note that though this prophylactic vaccine series has been successful in clinical trials, limited clinical success has been achieved for vaccines to treat already-contract HPV infections. This highlights the importance of prevention by administering HPV vaccines in the appropriate populations.^6^ Unfortunately, despite current recommendations and proven efficacy, vaccination rates in the US are drastically lower than rates of other childhood vaccines such as the measles, mumps, and rubella vaccine series.^1^ Low adherence to HPV vaccination may be influenced by the perception of HPV as a low health priority and vaccination as an elective health measure.^1^ Additionally, the lack of adequate information provided to both patients and their parents is a major barrier to obtaining an HPV vaccine.^8^

The lack of knowledge by patients, parents, and even healthcare providers is a general trend that could be addressed through education regarding HPV prevention and vaccination. An adolescent is more likely to get vaccinated if they have been educated on the benefits and risks, often through a healthcare provider.^9^ Primary care physicians, nurse practitioners, physician assistants, nurses, and athletic trainers (ATs) are healthcare providers working with this high-risk population, thus putting them in ideal positions to educate about HPV. ATs work in secondary school systems, college/university settings, industry, and medical settings. They are healthcare professionals who assist in the prevention, diagnosis, and treatment of injuries that occur to the physically active. Importantly, athlete refers to someone proficient or participating in sport or some form of physical activity.

Given ATs’ proximity to student-athletes, they may be in a position to impact their patients’ attitudes towards vaccinations and further educate them on the prevention and containment of HPV. The athlete, once educated, can then hypothetically inform others and protect themselves, sexual partners, and other contacts in turn helping to prevent HPV-related cancers. ATs can help create and facilitate community events and informative interactive interventions or specifically inquire about vaccinations when playing a role in the conduction of preparticipation physical exams.^10^ To our knowledge, no research exists about the knowledge, roles, and responsibilities of the AT population in educating athletes and organizations about the risks associated with HPV infection. This study aimed to investigate AT knowledge about HPV vaccination, as well as their perceived role and responsibility in HPV education. We hypothesize that overall ATs will have knowledge similar to the general population and that they could play a positive role in educating stakeholders.

## Methods

A cross-sectional survey was designed and utilized in this study. The use of cross-sectional survey with both quantitative and qualitative data collection has been previously been validated as a useful source of methodology by multiple research professionals.^11,12^ Additionally, the survey was piloted locally by 10 senior researchers and clinicians. The survey was sent to middle school, high school, and collegiate-level ATs through the National Athletic Trainers’ Association (NATA) data collection service program. It aimed to: (1) identify what ATs know about HPV, and (2) investigate ATs’ perceptions of their role and responsibility in educating about HPV. Institutional Review Board approval was obtained before releasing the survey.

First, study participants completed a consent form which indicated that participation was entirely voluntary with those enrolled holding the right to withdraw from the survey at any time without penalty. Second, subjects then completed 5 separate sections.

The first section consisted of demographic information: work settings, highest degree earned, and years certified. The second section assessed participants’ knowledge on HPV and the HPV vaccine. The third section consisted of perception-based questions that investigated subjects’ perceived role and responsibility in educating athletes about HPV and HPV vaccination. The fourth section was optional and indicated if the respondents had either been offered or received the HPV vaccine themselves or through their children. This was included to account for any personal biases in those answering the survey. The fifth subsection provided subjects with the opportunity to leave an open-ended response on ATs’ role in educating their patients about HPV and its prevention.

Survey respondents’ basic demographic, HPV vaccine status, participants’ knowledge, and their attitudes toward HPV infections were summarized using descriptive statistics (means and standard deviations (SDs) for quantitative variables and counts and proportions for categorical variables). For the bivariate analysis, a *t*-test and chi-squared calculations for parametric data, and the Wilcoxon Rank-Sum test or Fisher exact test for nonparametric were used to determine the associations between the independent variables and training received, any personal history of vaccination experience, and the highest degree earned by ATs, separately. All analyses were conducted using the SAS statistical software package (version 9.4; SAS Institute, Inc., Cary, NC, USA). All tests were 2-sided, and the statistical significance level used was .05. Of note, a power calculation was not utilized during the study. The sample size was instead determined based on the exploratory nature of the survey and novelty of all data collected similar to prior survey research.^13^

## Results

In total, 9000 surveys were sent out, and 460 ATs participated in the survey (5.11% response rate). Of those completing the demographics portion, a majority (59%; (252 out of 427)) under the age of 40 years (Table 1). Though a smaller portion of the overall survey population completion participated in the study, this still represented a diverse range of ATs as nearly every US state was represented, with only Alaska and Arkansas having no representation among the ATs partaking in the study. The highest proportion (48%; 208 of 427) of participants reported over 12 years of AT credentialing, and over 67% (290 of 427) of participants had at least a master’s degree. Table 2 reports ATs’ knowledge levels regarding HPV and HPV vaccination. Most ATs correctly identified that HPV could present asymptomatically (83% correct (355 of 402)) and that women are not the only patients at risk of HPV infection (95.8% correct (385 of 402)). Less than half of AT subjects knew that the HPV vaccine prevents 99% of cervical cancer (44.6% correct (176 of 401)), that the HPV vaccine is effective for a lifetime (43.0%; (173 of 402)), and that nearly 1 in 4 people in the US are currently infected with HPV (47.8% correct (192 of 402)). Furthermore, those ATs with previous HPV vaccination training had a statistically higher level of correct answers regarding HPV infection’s potential to be asymptomatic (*P* < .01) when compared to those with no prior training. Those with previous training had a higher rate of correctly answering 8 out of 11 knowledge-based questions.

**Table 1.** Athletic Trainer Demographics.

Subcategories	Total (%)
ATs age groups	
18-29 years old	139 (32.6)
30-39 years old	113 (26.5)
40-49 years old	78 (18.3)
50-59 years old	77 (18.0)
60+ years old	20 (4.7)
ATs gender	
Female	285 (66.6)
Male	141 (32.9)
Other	1 (0.2)
Prefer not to say	1 (0.2)
How many years have ATs held credential?	
0-6 years	140 (32.8)
7-12 years	81 (19.0)
More than 12 years	206 (48.2)
What is the highest degree ATs have earned?	
Academic doctoral degree (eg, PhD, EdD)	32 (7.5)
Bachelor’s degree	102 (23.9)
Clinical doctoral degree (eg, DAT, MD, and DO)	25 (5.9)
Master’s degree	265 (62.1)
Other	3 (0.7)
Each AT’s primary work setting	
College/University	165 (38.6)
Other	14 (3.3)
Secondary School-High School	183 (42.8)
Secondary School-Middle and High School	61 (14.3)
Secondary school-Middle School	5 (1.2)
Participate in a follow-up interview about the role and responsibilities of ATs regarding HPV?	
No	277 (64.9)
Yes	150 (35.1)

Each athletic trainer participating in the study completed a survey portion outlining their respective demographics as highlighted in Table 1.

**Table 2.** Athletic Trainer Responses to Knowledge-based Questions.

Question	True	False	Do not know
#1. HPV infection may be asymptomatic, n (%)	**355 (88.3)**	8 (2.0)	39 (9.7)
#2. Only women are at risk for HPV infection, n (%)	5 (1.2)	**385 (95.8)**	12 (3.0)
#3. HPV infection can cause genital warts, n (%)	**270 (67.2)**	16 (4.0)	116 (28.9)
#4. HPV infection can cause cervical cancer, n (%)	**366 (91.0)**	3 (0.7)	33 (8.2)
#5. The HPV vaccine prevents 99% of cervical cancer, n (%)	**176 (43.9)**	46 (11.5)	179 (44.6)
#6. The HPV vaccine has the potential to prevent more than 90% of HPV-attributable cancers, n (%)	**268 (67.0)**	5 (1.3)	127 (31.8)
#7. HPV is a sexually transmitted virus, n (%)	**319 (79.6)**	44 (11.0)	38 (9.5)
#8. Test performed to screen for cervical cancer is known as a blood smear test, n (%)	62 (15.5)	**182 (45.4)**	157 (39.2)
#9. Initial vaccination with HPV vaccine is recommended to girls and boys ages 9-11 years, n (%)	**310 (77.1)**	17 (4.2)	75 (18.7)
#10. The HPV vaccine is effective for a lifetime, n (%)	**173 (43.0)**	34 (8.5)	195 (48.5)
#11. Nearly 1 in 4 people are currently infected with HPV in the United States, n (%)	192 (47.8)	21 (5.2)	189 (47.0)

Each AT completed a second section of their survey which highlighted their respective level of knowledge related to HPV and HPV vaccination through 11 knowledge-based questions. The correct answer is bolded for each respective question listed in the table above.

Subjects were also asked personal questions regarding their own experience with either themselves or their children being offered and/or receiving the HPV vaccine. The results can be seen in Table 3, with each AT stratified by their knowledge score and their answer to each personal-based question. “Knowledge score” was calculated by the summation of each respective AT’s correct answer within the “knowledge-based question” portion of the survey. Table 3 presents evidence of a higher average knowledge score for the ATs answering “yes” to each question related to the HPV vaccine. The elevation in knowledge score was statistically significant for those ATs who had been offered the HPV vaccine (*P* < .001) and those who had received 1 or more doses of the vaccine (*P* < .001). Similarly, those ATs with any form of HPV vaccination training (268 out of 460) had a statistically significant increase in the personal history of receiving 1 or more vaccine doses (*P* = .004). Additionally, 48.5% of those ATs with a history of training had received a dose of the HPV vaccine themselves compared to only 33.1% of those who had never completed any form of HPV vaccination training.

**Table 3.** AT Personal Experience Stratified by Knowledge Score.

Personal questions	Knowledge score (no)	Knowledge score (yes)	*P* value*
Has your child/children been offered the HPV vaccine?	6.71	6.75	.947
Have you been offered the HPV Vaccine?	6.74	7.55	<.0001
Have you received 1 or more doses of the HPV Vaccine?	6.82	7.59	.0001
Do you have a child/children between the ages of 9 and 30 years?	7.25	6.85	.076
Has your child/children received 1 or more doses of the HPV Vaccine?	6.85	6.73	.785

Table 3 AT personal experience with HPV vaccination stratified by their knowledge-based questions.

Table 4 represents the relationship between ATs’ personal experience with HPV vaccination and their perception of various aspects of an AT’s role in the healthcare of their athletes. A positive personal history of HPV vaccination experience referred to those ATs with a history of being offered the HPV vaccine and/or having received at least 1 dose of the vaccine. In general, an AT’s personal vaccination experience did not have a statistically significant effect on their perception. However, a greater percentage of ATs with previous HPV vaccination experience (71.8%; 201 of 280) believed that ATs are responsible for general medical conditions affecting their athletes when compared to those without personal HPV vaccination experience (66.3%; 67 of 101) overall (*P* = .03).

**Table 4.** AT Personal History Regarding Vaccination Experience in Relation to Their Perception of Their Role in HPV Prevention.

Question and response	Any personal history of vaccination experience		*P*-value
	No	Yes	
	(N = 162) (%)	(N = 298) (%)	
Athletic trainers play a role in educating stakeholders about HPV			.404
Agree	36 (35.6)	100 (35.7)	
Disagree	19 (18.8)	58 (20.7)	
I prefer not to respond	1 (1.0)	0 (0.0)	
Neutral	45 (44.6)	122 (43.6)	
Educating athletes about HPV is important			.200
Agree	87 (86.1)	241 (86.1)	
Disagree	1 (1.0)	10 (3.6)	
I prefer not to respond	1 (1.0)	0 (0.0)	
Neutral	12 (11.9)	29 (10.4)	
ATs should be allowed to give HPV vaccinations under the direction of a physician			.420
Agree	13 (12.9)	36 (12.9)	
Disagree	57 (56.4)	157 (56.1)	
I prefer not to respond	1 (1.0)	0 (0.0)	
Neutral	30 (29.7)	87 (31.1)	
ATs should not educate athletes about HPV			.090
Agree	10 (10.0)	15 (5.4)	
Disagree	65 (65.0)	177 (63.4)	
I prefer not to respond	1 (1.0)	0 (0.0)	
Neutral	24 (24.0)	87 (31.2)	
I have talked to my supervising physician about HPV			.325
Agree	5 (5.0)	7 (2.5)	
Disagree	90 (89.1)	246 (87.9)	
I prefer not to respond	2 (2.0)	4 (1.4)	
Neutral	4 (4.0)	23 (8.2)	
My role in educating athletes about HPV is important			.210
Agree	31 (30.7)	88 (31.4)	
Disagree	25 (24.8)	44 (15.7)	
I prefer not to respond	1 (1.0)	2 (0.7)	
Neutral	44 (43.6)	146 (52.1)	
ATs have a societal responsibility to educate athletes about HPV			.276
Agree	28 (27.7)	72 (25.8)	
Disagree	30 (29.7)	73 (26.2)	
I prefer not to respond	1 (1.0)	0 (0.0)	
Neutral	42 (41.6)	134 (48.0)	
ATs are responsible for general medical conditions affecting their athletes			.030
Agree	67 (66.3)	201 (71.8)	
Disagree	13 (12.9)	15 (5.4)	
I prefer not to respond	2 (2.0)	1 (0.4)	
Neutral	19 (18.8)	63 (22.5)	
ATs should recommend HPV vaccination to their athletes			.709
Agree	27 (26.7)	69 (24.6)	
Disagree	28 (27.7)	76 (27.1)	
I prefer not to respond	3 (3.0)	4 (1.4)	
Neutral	43 (42.6)	131 (46.8)	
ATs can play a role in encouraging their athletes to receive the HPV vaccine			.930
Agree	61 (60.4)	161 (57.5)	
Disagree	12 (11.9)	33 (11.8)	
I prefer not to respond	1 (1.0)	2 (0.7)	
Neutral	27 (26.7)	84 (30.0)	
ATs should assess if their patients have received the tdap vaccine			.270
Agree	39 (38.6)	121 (43.4)	
Disagree	27 (26.7)	51 (18.3)	
I prefer not to respond	1 (1.0)	1 (0.4)	
Neutral	34 (33.7)	106 (38.0)	
ATs should recommend the influenza vaccine to their patients			.182
Agree	41 (40.6)	123 (43.9)	
Disagree	25 (24.8)	46 (16.4)	
I prefer not to respond	2 (2.0)	2 (0.7)	
Neutral	33 (32.7)	109 (38.9)	
ATs should assess the vaccine status of their patients during pre-participation physical exams			.093
Agree	45 (44.5)	145 (51.8)	
Disagree	24 (23.8)	43 (15.4)	
I prefer not to respond	2 (2.0)	1 (0.4)	
Neutral	30 (29.7)	91 (32.5)	

Table 4 highlights the relationship between AT participants’ personal history of HPV vaccination and their respective perceptions of the role of athletic trainers within HPV prevention practices.

AT perception of their role in HPV vaccination was further investigated with consideration for each AT’s respective educational background. As seen in Table 5, there was a statistically significant elevation in participants who felt that they should play a role in educating stakeholders about HPV when looking at those ATs who had obtained an academic doctoral degree compared to other respective levels of education (*P* = .049). For example, A majority of ATs in each educational group felt that ATs should assess if their student-athletes have received a Tdap vaccine, with 160 ATs in favor of assessing this status throughout all educational groups and only a total of 78 ATs opposed (*P* = .01). Finally, over half of the ATs in each academic category either agreed or were neutral when asked if ATs should assess the vaccine status of their patients during pre-participation physical exams (*P* = .048).

**Table 5.** AT Educational History Related to their Perception of their Role in HPV Prevention.

	What is the highest degree ATs have earned?					*P*-value
	Academic doctoral degree (eg, PhD, EdD)	Bachelor’s degree	Clinical doctoral degree (eg, DAT, MD, DO)	Master’s degree	Other	
Athletic trainers play a role in educating stakeholders about HPV, n (%)						.049
Agree	17 (60.7)	20 (22.7)	14 (58.3)	84 (35.4)	1 (33.3)	
Disagree	4 (14.3)	23 (26.1)	2 (8.3)	47 (19.8)	1 (33.3)	
I prefer not to respond	0 (0.0)	0 (0.0)	0 (0.0)	1 (0.4)	0 (0.0)	
Neutral	7 (25.0)	45 (51.1)	8 (33.3)	105 (44.3)	1 (33.3)	
Educating athletes about HPV is important, n (%)						.691
Agree	26 (92.9)	73 (83.0)	22 (91.7)	205 (86.5)	2 (66.7)	
Disagree	2 (7.1)	3 (3.4)	0 (0.0)	6 (2.5)	0 (0.0)	
I prefer not to respond	0 (0.0)	0 (0.0)	0 (0.0)	1 (0.4)	0 (0.0)	
Neutral	0 (0.0)	12 (13.6)	2 (8.3)	25 (10.5)	1 (33.3)	
ATs should be allowed to give HPV vaccinations under the direction of a physician, n (%)						.019
Agree	8 (28.6)	5 (5.7)	7 (29.2)	28 (11.8)	1 (33.3)	
Disagree	12 (42.9)	61 (69.3)	8 (33.3)	130 (54.9)	2 (66.7)	
I prefer not to respond	0 (0.0)	0 (0.0)	0 (0.0)	1 (0.4)	0 (0.0)	
Neutral	8 (28.6)	22 (25.0)	9 (37.5)	78 (32.9)	0 (0.0)	
ATs should not educate athletes about HPV, n (%)						.416
Agree	1 (3.6)	6 (6.9)	2 (8.3)	16 (6.8)	0 (0.0)	
Disagree	23 (82.1)	45 (51.7)	17 (70.8)	154 (65.3)	2 (66.7)	
I prefer not to respond	0 (0.0)	0 (0.0)	0 (0.0)	1 (0.4)	0 (0.0)	
Neutral	4 (14.3)	36 (41.4)	5 (20.8)	65 (27.5)	1 (33.3)	
I have talked to my supervising physician about HPV, n (%)						.427
Agree	0 (0.0)	3 (3.4)	0 (0.0)	9 (3.8)	0 (0.0)	
Disagree	23 (82.1)	79 (89.8)	22 (91.7)	209 (88.2)	2 (66.7)	
I prefer not to respond	0 (0.0)	1 (1.1)	1 (4.2)	4 (1.7)	0 (0.0)	
Neutral	5 (17.9)	5 (5.7)	1 (4.2)	15 (6.3)	1 (33.3)	
My role in educating athletes about HPV is important, n (%)						.083
Agree	11 (39.3)	21 (23.9)	15 (62.5)	72 (30.4)	0 (0.0)	
Disagree	2 (7.1)	19 (21.6)	2 (8.3)	44 (18.6)	1 (33.3)	
I prefer not to respond	0 (0.0)	0 (0.0)	0 (0.0)	3 (1.3)	0 (0.0)	
Neutral	15 (53.6)	48 (54.5)	7 (29.2)	118 (49.8)	2 (66.7)	
ATs have a societal responsibility to educate athletes about HPV, n (%)						.037
Agree	11 (39.3)	16 (18.2)	14 (58.3)	58 (24.6)	1 (33.3)	
Disagree	6 (21.4)	32 (36.4)	3 (12.5)	60 (25.4)	1 (33.3)	
I prefer not to respond	0 (0.0)	0 (0.0)	0 (0.0)	1 (0.4)	0 (0.0)	
Neutral	11 (39.3)	40 (45.5)	7 (29.2)	117 (49.6)	1 (33.3)	
ATs are responsible for general medical conditions affecting their athletes, n (%)						.583
Agree	22 (78.6)	54 (61.4)	21 (87.5)	167 (70.5)	3 (100.0)	
Disagree	2 (7.1)	10 (11.4)	0 (0.0)	16 (6.8)	0 (0.0)	
I prefer not to respond	0 (0.0)	1 (1.1)	0 (0.0)	2 (0.8)	0 (0.0)	
Neutral	4 (14.3)	23 (26.1)	3 (12.5)	52 (21.9)	0 (0.0)	
ATs should recommend HPV vaccination to their athletes, n (%)						.014
Agree	12 (42.9)	11 (12.5)	9 (37.5)	64 (27.0)	0 (0.0)	
Disagree	4 (14.3)	38 (43.2)	4 (16.7)	57 (24.1)	1 (33.3)	
I prefer not to respond	0 (0.0)	2 (2.3)	0 (0.0)	5 (2.1)	0 (0.0)	
Neutral	12 (42.9)	37 (42.0)	11 (45.8)	111 (46.8)	2 (66.7)	
ATs can play a role in encouraging their athletes to receive the HPV vaccine, n (%)						.690
Agree	19 (67.9)	47 (53.4)	17 (70.8)	136 (57.4)	2 (66.7)	
Disagree	1 (3.6)	16 (18.2)	3 (12.5)	25 (10.5)	0 (0.0)	
I prefer not to respond	0 (0.0)	1 (1.1)	0 (0.0)	2 (0.8)	0 (0.0)	
Neutral	8 (28.6)	24 (27.3)	4 (16.7)	74 (31.2)	1 (33.3)	
ATs should assess if their patients have received the tdap vaccine, n (%)						.007
Agree	20 (71.4)	30 (34.1)	13 (54.2)	97 (41.1)	0 (0.0)	
Disagree	3 (10.7)	29 (33.0)	4 (16.7)	42 (17.8)	0 (0.0)	
I prefer not to respond	0 (0.0)	0 (0.0)	0 (0.0)	2 (0.8)	0 (0.0)	
Neutral	5 (17.9)	29 (33.0)	7 (29.2)	95 (40.3)	3 (100.0)	
ATs should recommend the influenza vaccine to their patients, n (%)						.065
Agree	17 (60.7)	27 (30.7)	14 (58.3)	104 (43.9)	1 (33.3)	
Disagree	1 (3.6)	27 (30.7)	3 (12.5)	40 (16.9)	0 (0.0)	
I prefer not to respond	0 (0.0)	1 (1.1)	0 (0.0)	3 (1.3)	0 (0.0)	
Neutral	10 (35.7)	33 (37.5)	7 (29.2)	90 (38.0)	2 (66.7)	
ATs should assess the vaccine status of their patients during pre-participation physical exams, n (%)						.048
Agree	20 (71.4)	33 (37.5)	18 (75.0)	117 (49.4)	1 (33.3)	
Disagree	2 (7.1)	22 (25.0)	2 (8.3)	40 (16.9)	1 (33.3)	
I prefer not to respond	0 (0.0)	2 (2.3)	0 (0.0)	1 (0.4)	0 (0.0)	
Neutral	6 (21.4)	31 (35.2)	4 (16.7)	79 (33.3)	1 (33.3)	

Table 5 focuses on the perceived role of ATs in HPV prevention practices with consideration for general education background categories as listed above.

One other important aspect of our studies involved qualitative responses regarding ATs’ opinions on the topic at hand. Many study participants noted that though they did not feel it was an AT’s specific job to help an athlete decide on their vaccination status, but they agreed that an AT has a responsibility to educate their student-athletes on general medical conditions. One AT stated: “ I feel that ATs have a responsibility to educate and discuss all health concerns. Ultimately it is between the parent and the athlete to make a decision. After the decision is made, it is the ATs place to respect the decision and not judge.” This idea came up from numerous subjects when answering the qualitative portion of the survey. Many ATs also noted legislative or cultural pressures that could be barriers within their working environment. One AT summed this feeling up well, stating that “talking with a student athlete about HPV might get one in some serious trouble at our facility. [This] does not mean ATs should not know about it or discuss it during the course of a relevant evaluation.” Finally, a number of ATs also noted that they did not feel that ATs should be responsible for educating their athletes on HPV, citing equally-important factors such as “burn out which already exists within the profession itself.”

## Discussion

Our survey provides insight into ATs’ knowledge and confirms our hypothesis that further education on this subject may be beneficial. Nearly 1 in 4 people are infected with HPV, highlighting the importance of identifying any potential avenues for increasing HPV vaccination and prevention.^1^ However, for ATs to be able to educate their athletes on topics like HPV prevention, they must first be educated on the topic themselves. As shown in this study, ATs with previous HPV training and education had a greater rate of answering the knowledge-based questions correctly. Previous literature has identified the impact of educational interventions within the field of athletic training.^14,15^ A 2014 study by Welch et al presented evidence that educational modules effectively increased ATs’ knowledge of utilizing evidence-based practices. Suggesting a need for increased education regarding HPV vaccination and prevention if we were to begin further involving ATs in HPV prevention. This is further supported by the data collected in this research, as 192 of the 460 ATs participating in the study reported no history of any form of HPV vaccination training.

Additionally, Table 4 provides evidence that an AT’s personal experience with HPV prevention also impacts their perception of their role in prevention within their respective student-athlete populations. Those with previous personal HPV vaccination experience were significantly more likely to feel that ATs are responsible for the general medical conditions affecting their athletes. However, both ATs with and without this experience felt that educating athletes about HPV is important and largely agreed that ATs should educate stakeholders about HPV.

One evident point was the effect of confounding factors on whether ATs felt they could rightly involve themselves in HPV education. For example, many ATs used the open-ended portion of the survey to indicate that the environment in which they practiced may inhibit their ability to participate in HPV prevention discussions. One AT stated that because they practice in a particular state, they are prohibited from discussing topics like sexually transmitted diseases such as HPV within their work setting. Other ATs mention that working at the high school level presents a challenge when discussing sexually related diseases like HPV with minors. Another AT noted that though they “feel that an AT should provide education to athletes in all areas of health,” their work environment (a private high school setting) does not allow for this. This has been an important topic discussed by other research works in the past as well, dating back to 2016.^16^ This study indicated that, though a number of states had attempted to implement jurisdiction in favor of HPV vaccination, only Rhode Island was successful in doing so. The ensuing years after the failed jurisdiction have continued to impose difficulty barriers to healthcare allies being able to safely advocate for HPV vaccination in their patients as evidenced by the qualitative responses noted here in the present study.

ATs also commented they don’t feel responsible for treating the general medical conditions of their patients, but that they should be equipped with the knowledge needed to educate their athletes on disease prevention when applicable. Furthermore, many of those who felt educating about HPV was out of the AT scope also felt that it would benefit their athletes if ATs worked closely with school nurses to assist and monitor those who may have questions regarding vaccinations. Finally, 1 response summed up the view of a large group of ATs with a more neutral response to our survey. One subject noted that “it is my responsibility as an AT to make sure that correct information is provided to my student population, so families can make their own decisions on vaccination use or not. My role is a support person for the families.” This largely aligns with the intention of the study at hand. The intention was not to present another task that ATs should be responsible for, but rather to identify another area of medical practice in which education can be provided to student-athletes and other stakeholders which would allow them to make better-educated decisions regarding topics such as HPV vaccination.

An important point regarding AT educational experience arises when considering education experience alongside ATs’ perception of their role in HPV prevention. A significantly higher percentage of ATs with either an academic (60.7%) or clinical doctorate (58.3%) felt that ATs have a societal responsibility to educate athletes when compared to ATs with either a bachelor’s (22.7%) or master’s (35.4%) degree (*P* = .049). Finally, given that many ATs in each academic subgroup felt that ATs should assess the vaccine status of their patients during pre-participation physical exams, there is evidence to suggest a role for ATs to play in vaccine education. Considering the increased support for assessing Tdap status when compared to HPV vaccine status, the larger issue at hand may be the overall public perception of HPV prevention and education. For example, a 2014 study showed that parental concerns regarding the HPV vaccination largely mirrored fear regarding other similar vaccines, indicating that proper education by physicians may help allay many of the existing public fears and concerns.^17^

Many previous studies have suggested that educational interventions regarding HPV prevention can decrease public fear and confusion regarding HPV vaccination.^18-20^ This presents an important future direction for research in which an educational module or session may be provided to ATs regarding HPV prevention and vaccination, followed by a similar survey to identify their perception of their role in prevention. Future research should also investigate the potential for AT involvement in educating their athletes and other stakeholders on other areas of general disease prevention, given that the majority of study participants did feel they were responsible for the general well-being of their athletes.

Other important future directions for researchers to investigate may involve the impact of vaccine perceptions and hesitancy within different settings, something that was not fully explored. Furthermore, utilizing geographical breakdown such as zip codes may provide additional understanding of where vaccine education efforts should further be focused.

## Limitations

Some limitations in the study certainly exist. Because a survey method was used to collect data, the results are subject to both sampling bias and an inability to account for incorrectly answered questions by study subjects. The NATA database was utilized to account for sampling bias as it did provide access to ATs nearly nationwide. However, our data was subject to potential response bias, given it relied upon the truthful responses by participants who answered questions on their own with no supervision. As previously referenced, the current “taboo” nature of discussing sexually transmitted diseases such as HPV limited the extent of ATs who felt they could safely contribute to education and discussion of this topic with their patients. Future research involving different disease prevention etiologies such as influenza or tetanus vaccinations, may be able to account for a broader depiction of ATs’ perception of their role in the healthcare of their athletes specific to vaccine-preventable diseases.

Additionally, many ATs responded to our questions with either “prefer not to say” or “neutral” answers. These options, while both valid and important, may limit the number of true responses that ATs provided to “agree or disagree” questions. We also did not complete a formal, robust qualitative survey to accompany the open-ended responses that many ATs provided. This may limit the impact of the qualitative responses we gathered and indicate another future direction for this area of research. Finally, it is imperative to address the low response rate (5.11%) received from the total number of surveys distributed within the study. To mitigate non-response rate bias from this lower response rate as best as possible, our study utilized both qualitative and quantitative data to truly obtain insight into why the ATs participating in the study answered the ways that they did. Other notable studies have been seen to provide valuable insight despite lower response rates.^13^ As noted above, this does highlight the need for further investigation regarding ATs’ experiences related to the topic at hand. Further research would allow for increased generalizability which may be deemed difficult with a lower response such the one found in the research at hand.

## Conclusions

HPV-related disease is a common preventable medical condition seen within the patient population that ATs treat. As such, ATs, as allied health care providers, are well positioned to educate stakeholders on HPV prevention. However, overall knowledge levels are low within the AT community. If ATs were engaged in educating about HPV vaccination and prevention, this would require a path for ATs to gain that education, most likely through collaboration with their supervising physician. This is the first attempt to understand how to utilize ATs as an important partner in a novel approach to advocating and educating for HPV prevention. This advocacy could increase vaccination rates, thus helping to decrease HPV-related infections and subsequent cervical cancers in the student-athlete population. As such, the potential impact of utilizing ATs within HPV education provides a promising approach which requires further research and training to be provided to said athletic trainers moving forward.
